# Detection of Orange Essential Oil, Isopropyl Myristate, and Benzyl Alcohol in Lemon Essential Oil by FTIR Spectroscopy Combined with Chemometrics

**DOI:** 10.3390/foods10010027

**Published:** 2020-12-24

**Authors:** Nur Cebi, Osman Taylan, Mona Abusurrah, Osman Sagdic

**Affiliations:** 1 Department of Food Engineering, Faculty of Chemical and Metallurgical Engineering, Yıldız Technical University, 34210 İstanbul, Turkey; sagdic@gmail.com; 2 Department of Industrial Engineering, Faculty of Engineering, King Abdulaziz University, Jeddah 21589, Saudi Arabia; otaylan@kau.edu.sa; 3 Department of Management Information Systems, College of Business Administration, Taibah University, Madinah 42353, Saudi Arabia; mabusurrah@taibahu.edu.sa

**Keywords:** FTIR, lemon essential oil, PLSR, PCR, HCA, adulteration, chemometrics

## Abstract

Essential oils are high-valued natural extracts that are involved in industries such as food, cosmetics, and pharmaceutics. The lemon essential oil (LEO) has high economic importance in the food and beverage industry because of its health-beneficial characteristics and desired flavor properties. LEO, similar to other natural extracts, is prone to being adulterated through economic motivations. Adulteration causes unfair competition between vendors, disruptions in national economies, and crucial risks for consumers worldwide. There is a need for cost-effective, rapid, reliable, robust, and eco-friendly analytical techniques to detect adulterants in essential oils. The current research developed chemometric models for the quantification of three adulterants (orange essential oil, benzyl alcohol, and isopropyl myristate) in cold-pressed LEOs by using hierarchical cluster analysis (HCA), principal component regression (PCR), and partial least squares regression (PLSR) based on FTIR spectra. The cold-pressed LEO was successfully distinguished from adulterants by robust HCA. PLSR and PCR showed high accuracy with high R^2^ values (0.99–1) and low standard error of cross-validation (SECV) values (0.58 and 5.21) for cross-validation results of the raw, first derivative, and second derivative FTIR spectra. The findings showed that FTIR spectroscopy combined with multivariate analyses has a considerable capability to detect and quantify adulterants in lemon essential oil.

## 1. Introduction

Essential oils are natural lipidic substances extracted from fruits, vegetables, and spices, and they are used in many sectors throughout the whole world due to their unique pure and characteristic functional properties [1]. Flavor, fragrances, cosmetics, aromatherapy, and phytomedicine industries demand essential oils because of their unique characteristic properties [2]. Previous studies have shown that citrus essential oils have been used as natural preservatives, flavorings, antioxidants, antibacterial, and antifungal agents in various foods [3]. Citrus oils have found applications in the food sector throughout the world as flavoring agents in a wide variety of foodstuffs, such as beverages and confectionary, since they were announced as “generally recognized as safe” by food regulations.

Lemon essential oil is highly demanded in the food and beverage industry because of its health-beneficial characteristics (sedative, anxiolytic, antidepressant, and antispasmodic effects) and intrinsic flavor properties [4]. Lemon oil is used as an ingredient in body care products, cosmetics, and medications because of its desirable characteristic properties [5].

From an economic point of view, lemon essential oil as a high-value natural product has high economic importance worldwide with the exports and imports between countries. The price of authentic lemon essential oil is relatively high; thus, authentic lemon essential oil is prone to be adulterated through economic motivations. Adulteration causes unfair competition between vendors, disruptions in national economies, and crucial risks for consumers throughout the world [6]. Determination of the authenticity of essential oils such as lemon essential oil has crucial importance in terms of consumers, producers, importers, and exporters [7]. Previous studies showed that lemon essential oil was adulterated with cheaper sweet-orange oil and diluents such as benzyl alcohol, benzyl benzoate, isopropyl myristate, and phthalate esters, etc. [7,8]. The quality properties of lemon essential oil were defined by the international standard of lemon oil [9]. This standard defined the lemon essential oil as expression of the fresh fruit of Citrus limon without the aid of heat and with or without previous separation of the pulp and the pee [9]. Cold-pressed lemon oils are mainly used in cosmetic, perfume, pharmaceutical and food and beverage industries, and various cold-pressed extraction processes may be used for the production of lemon oil [10]. According to the standard, the quality of lemon oil was determined by the assessment of a lot of parameters such as appearance, color, odor, relative density, refractive index, optical rotation, acid value, carbonyl value and chromatographic profile. However, there is a need for rapid, strong, effective and reliable methodologies for the determination of the authenticity or adulteration of lemon essential oil. So far, researchers have performed quite limited studies on the adulteration of lemon essential oil. Adulteration of lemon essential oil was evaluated by using gas chromatography–combustion–isotope ratio mass spectrometry (GC–C–IRMS) and Gas Chromatography techniques in previous studies [8,10,11]. As an alternative technique, FTIR (Fourier transform infrared spectroscopy) can be used for the determination of the adulteration of lemon essential oil. Fourier transform infrared spectroscopy is known as a rapid, non-destructive, reliable, effective, and low-cost analytical technique that provides fingerprint information about the chemical structure of materials [12]. Up to now, FTIR spectroscopy combined with chemometrics has been used for the determination of adulterants in various food matrixes [13]. In addition, chemical composition or components of various essential oils were effectively evaluated by using FTIR spectroscopy [14,15,16,17]

The aim of this research was to detect three different adulterants in cold-pressed lemon essential oil by using FTIR spectroscopy in combination with chemometrics of HCA (hierarchical cluster analysis), PLSR (partial least squares regression), and PCR (principal component regression). To the best of our knowledge, this study is the first attempt for the determination of sweet orange oil, BnOH (benzyl alcohol), and IPM (isopropyl myristate) adulteration in a lemon essential oil utilizing FTIR technique combined with multivariate statistics. The safety assessment of benzyl alcohol and isopropyl myristate was reported in previous publications; isopropyl myristate and benzyl alcohol were reported to be safe as cosmetic ingredients [18,19]. However, detection of the dilution of lemon essential oil by IPM and BnOH is an important issue in terms of the authenticity of lemon essential oil.

## 2. Materials and Methods

### 2.1. Equipment

FTIR spectra were obtained by using Bruker Tensor 27 FTIR spectrometer (Bruker-Germany) in the spectral range of 400–4000 cm^−1^. FTIR spectrometer had an ATR (attenuated total reflectance) accessory with a diamond crystal. The commercial spectral library of Bruker (ATR FTIR Complete Library, composed of more than 20,000 reference spectra) was used for identity confirmation of used chemicals.

### 2.2. Essential Oils and Chemicals

The original cold-pressed lemon essential oils (*n* = 3) and cold-pressed orange essential oils (*n* = 3) were purchased from reliable (well-known) producer companies in Turkey. Benzyl alcohol (BnOH) and isopropyl myristate (IPM), with purity higher than 99%, were obtained from Zag Industrial chemicals (Turkey). Ethyl alcohol (Schuchardt, FRG, GC Merck > 98%) was used for cleaning diamond ATR crystal. Essential oils were stored at 4 °C prior to the FTIR analyses.

### 2.3. Sample Preparation

Three different cold-pressed lemon essential oils were coded as LEO_1_, LEO_2,_ and LEO_3_. Three different cold-pressed orange essential oils were coded as OEO_1_, OEO_2,_ and OEO_3_. Abbreviations of BnOH and IPM were used for benzyl alcohol and isopropyl myristate, respectively. LEO_1_, LEO_2,_ and LEO_3_ were spiked with OEO_1_, OEO_2,_ and OEO_3_, respectively, at the adulteration levels of 1%, 5%, 10%, 20%, 40%, and 50%. Additionally, three cold-pressed lemon essential oils (LEO_1_, LEO_2_, and LEO_3_) were spiked with BnOH and IPM at the concentrations of 1%, 5%, 10%, 20%, 40%, and 50%. In total, fifty-four adulterated samples were prepared for FTIR analyses. Samples were stored in amber glass vials (1.5 mL) prior to the spectral measurements. Additionally, an authentic cold-pressed lemon essential oil was purchased from the producer and separately spiked with OEO_1_, BnOH and IPM at the concentration of 1%, 4%, 8%, 16% and 32% (*v/v*) to test calibration models.

### 2.4. FTIR Measurements

All samples were kept at room temperature (25 °C) for 30 min prior to the FTIR analyses. An ATR accessory (single bounce) was used in all spectral acquisition. Spectral measurement parameters of resolution and accumulation were selected as 4 cm^−1^ and 16 scans, respectively. OPUS program Version 7.2 (Bruker Gmbh) was used for instrument control and data acquisition. Each sample was placed on a diamond ATR crystal with the help of a Pasteur pipette. The ATR crystal was cleaned with ethanol (80% *v/v*) prior to each spectral acquisition. The background air spectrum was scanned before each acquisition.

### 2.5. Chemometrics

#### 2.5.1. Hierarchical Cluster Analyses

Original cold-pressed lemon essential oils were discriminated from adulterated samples, orange essential oils, and chemicals on the basis of their FTIR spectra by using chemometrics of hierarchical cluster analysis. HCA analysis was conducted by using the chemometrics software OPUS Version 7.2 (Bruker, Germany). First derivative spectra of all samples were used for HCA through Ward’s algorithm and Euclidean distance. Spectral ranges of 1387–507 cm^−1^, 1528–1485 cm^−1^, 1772–1701 cm^−1,^ and 2878–2812 cm^−1^ were selected to discriminate original lemon essential oils from other samples in hierarchical cluster analysis.

#### 2.5.2. Quantification of Adulterants in the Lemon Essential Oils

The quantities of adulterants (orange essential oil, BnOH, and IPM) in the composition of lemon essential oil were predicted by the employment of the Grams IQ (Galactic Industries Corp, Salem, NH, USA) software for the adulteration levels of 1%, 5%, 10%, 20%, 40%, and 50%.

The first derivative and second derivative spectra were included in the PLSR (partial least squares regression) and PCR (principal component regression) multivariate analysis. Cross-validation curves were built at the concentration range of 0% and 100% for each adulterant. Cross-validation curves were built on the basis of selected spectral ranges of FTIR spectra. The spectral range should include information describing the concentration variation of the analyte or other matrix constituents [20]. In the present research, spectral ranges of 1666–1693 cm^−1^, 560–777 cm^−1^, and 1716–1755 cm^−1^ were selected for OEO, BnOH, and IPM, respectively.

## 3. Results

### 3.1. Characterization of Lemon Essential Oils by FTIR Spectroscopy

**Figure 1:**
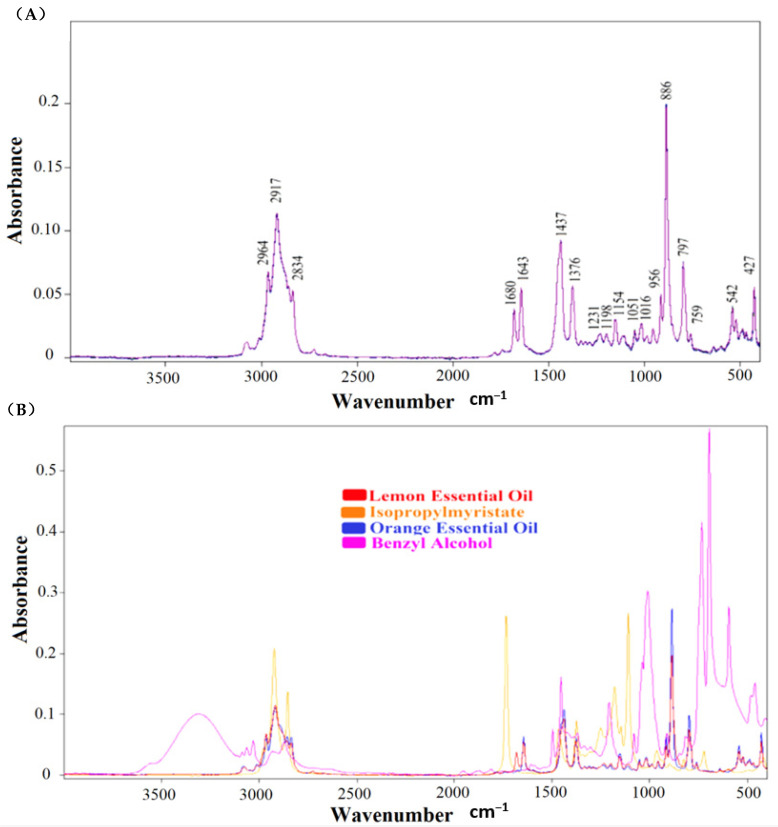
Overlapped FTIR spectra of cold-pressed lemon essential oils at the mid-infrared region (400–4000 cm^−1^) (**A**); FTIR spectra of lemon essential oil, orange essential oil, isopropylmyristate and benzyl alcohol (**B**).

Overlapped FTIR spectra of cold-pressed lemon essential oils (LEO_1_, LEO_2,_ and LO_3_) are presented in Figure 1A. FTIR spectrum of material includes the “fingerprint” chemical information, which is specific to investigated material [21]. In this way, the authenticity of materials could be determined by using the unique FTIR spectral data. Lemon essential oil is composed of terpenes (about 94% mainly (+)- limonene), sesquiterpenes, and aldehydes [22]. Identical spectral features were observed in the FTIR spectra of three cold-pressed lemon essentials, as can be seen in Figure 1A. Main peaks were observed at 2964, 2917, 2843, 1680, 1643, 1437, 1376, 1231, 1198, 1154, 1051, 1016, 956, 886, 797, 542 and 427 cm^−1^. The vibrational bands around ~2900 cm^−1^, ~1700 cm^−1,^ and ~1100 cm^−1^ may include spectral features arising from C-H, C=O, and C-O stretching vibrations of terpenoid components, respectively [23]. The peak around 2964 cm^−1^ corresponds to the –CH_3_ asymmetric and symmetric stretching vibrations [14]. The peaks at 2917 cm^−1^, 1680 cm^−1^, 1643 cm^−1^, 1437 cm^−1^, 1376 cm^−1^, 1154 cm^−1^, 886 cm^−1^, and 797 cm^−1^ correspond to the C-H stretching vibrations of alkanes, C=O stretching vibrations, C=C stretching vibrations of alkanes, C-H bending vibrations of alkanes, O.H. bending vibrations of phenols, C-O stretching vibrations of tertiary alcohols, C-H stretching vibrations of aromatics and C=C bending vibrations of alkanes, respectively [14,24]. Overlapped FTIR spectra of cold-pressed lemon essential oil, cold-pressed orange essential oil, isopropyl myristate, and benzyl alcohol are presented in Figure 1B. As can be seen, OEO and LEO have similar spectral properties when compared to chemicals of isopropyl myristate and benzyl alcohol. BnOH and IPM have significantly distinct spectral properties at the spectral range of 400–4000 cm^−1^.

### 3.2. Determination of Authentic Lemon Essential Oil by Hierarchical Cluster Analysis

**Figure 2:**
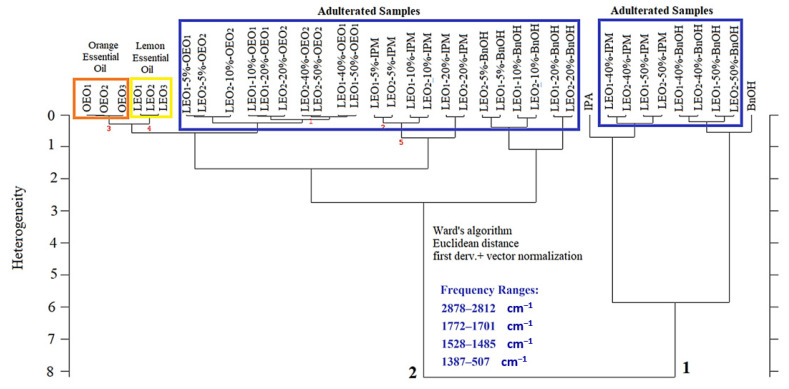
Two-dimensional hierarchical cluster analysis (HCA) plot of FTIR measurements from cold-pressed lemon essential oil, cold-pressed orange essential oil, adulterated samples, isopropyl myristate (IPM) and benzyl alcohol (BnOH).

Hierarchical cluster analysis (HCA) was performed for discrimination of authentic cold-pressed lemon essential oils from cold-pressed OEOs, adulterated samples (OEOs, BnOH, and IPM), BnOH, and IPM. HCA provides an opportunity for visualization of the hidden relationship between investigated samples by using 2-D plots (dendrograms), which presents a cluster pattern of investigated elements [25]. HCA was performed by using selected spectral ranges of 2878–2812 cm^−1^, 1772–1701 cm^−1^, 1528–1485 cm^−1^ and 1387–507 cm^−1^. First derivative spectra were processed through Ward’s algorithm and Euclidian distance. The euclidian distance was used for the calculation of spectral distances. Ward’s algorithm (minimum variance agglomeration) has a strong capability to obtain clear classification patterns; thus, Ward’s algorithm was used for lightening a lot of challenging adulteration and authenticity problems in previous studies [26,27,28,29,30]. The output of the hierarchical cluster analyses, a 2-D dendrogram, is presented in Figure 2. The whole sample set was initially clustered in “two” classes. LEO (*n* = 3) and OEO (*n* = 3) samples were significantly discriminated from other samples on the dendrogram. LEO and OEO samples were marked by using a yellow rectangle and a red rectangle, respectively. Although OEO and LEO samples have similar FTIR spectra, a clear classification of these samples was obtained without any false agglomeration. Additionally, chemicals of IPM and BnOH were classified distinctly from essential oils and adulterated samples on the right side of the HCA dendrogram. As can be seen, the highest BnOH and IPM spiked samples (40% and 50%) were classified closer to the IPM and BnOH samples. Overall, it can be concluded from the dendrogram that authentic cold-pressed lemon essential oil samples could be accurately distinguished from cold-pressed orange essential oil, adulterated samples, BnOH, and IPM.

### 3.3. Quantification of OEO, BnOH, and IPM in LEO by Using PLSR and PCR Models

Chemometrics of partial least squares regression (PLSR) and principal component regression (PCR) were used for quantification of cold-pressed OEO, BnOH, and IPM in cold-pressed lemon essential oil. Multivariate calibration methods are used to extract interesting information from high throughput analytical data [31]. PLSR and PCR models are used to obtain a linear relationship between the concentration and measured intensity of investigated compounds (constituents) by using instrumental data [32]. PLSR and PCR techniques are widely used in combination with vibrational spectroscopy for the determination of adulteration with high accuracy with the help of selection concentration-related spectral regions [33]. Basically, in the PLSR, the spectral information in the thousands of infrared frequency is reduced to several “latent variables”, which is crucial to explain the model’s variation [34]. Similarly, PCR is used with the aim of building calibration curves that correlate the concentration levels and the absorbance intensity of investigated constituents. PCR has been known as a practical approach; response variables are regressed with respect to the principal components of covariates [35].

**Figure 3:**
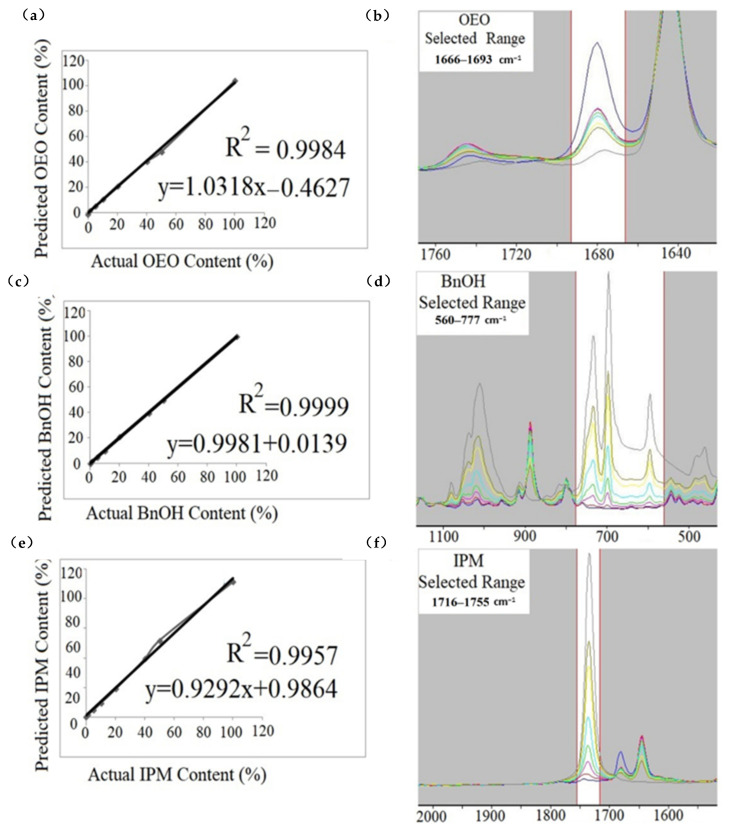
Cross-validation r (partial least squares regression (PLSR)—raw spectra): actual versus predicted values and selected calibration ranges (**a**) OEO adulteration in LEO, (**b**) selected calibration range for OEO adulteration in LEO, (**c**) BnOH adulteration in LEO, (**d**) selected calibration range for BnOH adulteration in LEO, (**e**) IPM adulteration in LEO, (**f**) selected calibration range for IPM adulteration in LEO.

PLSR and PCR, robust multivariate techniques, were successfully employed for quantification of various adulterants in complex food-related matrices in terms of evaluation of food authenticity and traceability [36]. PLSR and PCR analyses were performed by using raw, first-derivative, and second-derivative FTIR spectra of all samples. The different spectral range was selected for each adulterant type (OEO, BnOH, and IPM). Cross-validation curves (PLSR) and selected spectral ranges are presented in Figure 3. The spectral ranges of 1666–1693 cm^−1^, 560–777 cm^−1^, and 1716–1755 cm^−1^ were selected for the quantification of the adulterants OEO, BnOH, and IPM, respectively. Concentration levels were 0%, 1%, 5%, 10%, 20%, 40% and 50% (*v/v*) for each adulterant. The spectral range should include information describing the concentration variation of the analyte or other matrix constituents [20]. In the current study, the spectral region in which concentration change was clearly observed was selected for each adulterant. One can observe from the FTIR spectra that the intensities of concentration-related bands in the selected spectral regions clearly increased with the rising concentration of adulterants. Calibration and cross-validation equations, R^2^, LOGPress, SECV (standard error of cross-validation), and bias are presented in Table 1. The prediction ability of developed PLS and PCR models are evaluated on the basis of especially R^2^ and SECV values; the model that has the highest R^2^ values and lowest SECV values has the highest ability to describe the relationship between actual adulterant concentration and predicted adulterant concentration. The SEC is defined as the standard error of calibration and SEC is formulated as the square root of the residual variance divided by the number of degrees of freedom. The SECV value is defined as the standard error of cross-validation (prediction) [37]. In the present study, three latent variables were used for each model. Best SECV values were obtained when three latent variables were used.

Bias could be defined as the systematic error of the calibration or cross-validation and calculated as the average difference between the reference and predicted values [38]. As it can be seen in Table 1, quite fair R^2^ values were obtained in all cross-validation models. The determination coefficient (R^2^) changed at the range of 0.9902–1 and 0.9906–0.9999 in the calibration models and cross-validation models of the raw, first derivative, and second derivative FTIR spectra of samples, respectively. The determination coefficient (R^2^) normally changes between “0” and “1”. The closeness of the (R^2^) value to the “1” supports the reliability of the model since (R^2^) is a statistical measure of how close the data are to the fitted regression line. Additionally, calibration and cross-validation model equations are presented in Table 1; one can observe that the slope of the equations changes around “0.9” and “1”, this means that the obtained equations are quite similar to the equation of y = x. In a regression model, the predicted concentration of adulterant (y) is equal to the actual concentration (x) of adulterant when y = x; in other words, the regression model has the ability to predict adulteration concentration with 100% accuracy on the basis of FTIR spectral data. Additionally, SECV ranged between 0.58 and 5.21. The systematic error of cross-validation models altered around 0.27 and 4.85. All calibration and cross-validation models showed high accuracy when they were evaluated in terms of R^2^, SECV, and bias values. Results from the current research showed that developed PLSR and PCR models could be effectively used for the prediction of cold-pressed OEO, IPM, and BnOH adulteration in cold-pressed LEOs. Additionally, an authentic cold-pressed lemon essential oil was purchased from the producer and separately spiked with OEO_1_, BnOH and IPM at concentrations of 1%, 4%, 8%, 16% and 32% (*v/v*). Developed FTIR-PLSR and PCR models (raw spectra) were used for the quantification of adulterants. OEO_1_ concentrations were determined as 1.15%, 4.18%, 8.22%, 16.15% and 32.25% (*v/v*) by the PLSR model. BnOH concentrations were determined as 1.08%, 4.10%, 8.08%, 16.12% and 32.05% (*v/v*) by the PLSR model. IPM concentrations were determined as 1.18%, 4.31%, 8.14%, 16.22% and 32.16% (*v/v*) by the PLSR model. In addition, adulterant concentrations were quantified as 0% for authentic cold-pressed lemon essential oil by the PLSR model. Quite similar results were obtained by using the PCR models. These results showed the efficiency of FTIR spectroscopy in combination with chemometrics models of PLSR and PCR.

## 4. Discussion

In the current research, PLSR and PCR techniques were successfully employed for quantification OEO, BnOH, and IPM by using a robust vibrational technique, FTIR spectroscopy. To the best of our knowledge, this study is the first attempt for quantification of adulterants in LEO by using FTIR spectroscopy combined chemometrics of PLSR and PCR. Previous studies presented valuable results for the characterization of citrus essential oils such as lemon and orange oil [39]. In the present research, highest intensity was observed for the spectral band at 886 cm^−1^ and similar spectral properties were observed in the FTIR spectrum of lemon essential oil in previous research [39]. Additionally, quite limited studies were performed for the detection of adulteration in cold-pressed lemon essential oil. Schipilliti et al. (2012) utilized gas chromatography–combustion–isotope ratio mass spectrometry for the determination of the authenticity of cold-pressed lemon essential oils. They proved the effectiveness of stable isotope ratio analysis for the detection of genuine LEOs [8]. In another study, Lifshitz and Stepak (1969) determined the addition of orange oil, d-limonene, and commercial terpenes to the Israel lemon oil by using gas–liquid chromatography, and they detected the adulterants with a detection limit of 10% [11]. A previous study used the HPLC technique for the detection of low-quality lemon essential oil in cold-pressed LEO, and they detected adulteration concealer compounds in the investigated LEOs [40]. Techniques are reliable, robust, and widely used though they are generally arduous, time-consuming, and require toxic chemicals and expert operators. In this respect, FTIR spectroscopy can be chosen as an appealing alternative since it is a cost-effective, rapid, reliable, robust, eco-friendly analytical technique and has the capability to obtain the chemical fingerprint of materials in just a few minutes [41]. Previous contributions proved the effectiveness of vibrational spectroscopy techniques such as FTIR and Raman spectroscopy for the determination of the authenticity of essential oils [15,17,42,43,44]. Additionally, previous applications of FTIR spectroscopy in combination with chemometrics shed light on the quality and purity evaluation of essential oils [45]. No previous study has investigated the adulteration of LEOs. In this research, FTIR spectroscopy, in combination with multivariate statistics of PLSR and PCR, was effectively used for prediction of the adulteration levels of OEO, BnOH, and IPM adulterants in a few minutes with minimum sample preparation.

## 5. Conclusions

The current research presented an application of FTIR spectroscopy combined with chemometrics for quantification of adulterants (OEO, BnOH, and IPM) in LEOs. Results showed that adulterants were successfully quantified at the concentration range of 0–50% (*v/v*) by using PLSR and PCR. Additionally, HCA was effectively employed for discrimination of LEOs from adulterated samples by the 2-D plots (dendrograms) in which separate clusters and sub-clusters were clearly visualized on the basis of FTIR spectra. PLSR and PCR showed high accuracy with high R^2^ values (0.99–1) and low SECV values (0.58 and 5.21) for cross-validation results of the raw, first derivative, and second derivative FTIR spectra. Essential oils as natural extracts are prone to be adulterated through economic motivations. There is a need for cost-effective, rapid, reliable, robust, and easy-to-operate methodologies to maintain the quality of essential oils and tremendous products in which they involved. FTIR spectroscopy, in combination with multivariate analyses of PLSR, PCR, and HCA, showed high potential for detection of investigated adulterants and discrimination of natural cold-pressed lemon essential oil. Additionally, integration of the developed methodology to the hand-held FTIR spectrometers may help detection of frauds in the essential oil industry and whole supply chain. The findings from the current research may shed light on various adulteration incidents in which essential oils, foods, natural extracts, and high-value products are deteriorated.

## Figures and Tables

**Table 1:** PLSR and PCR results of calibration and cross-validation results of raw, first and second derivative FTIR spectra of adulterated lemon essential oils.

Samples	Calibration Technique	Spectra	Equation		*R* ^2^		LOGPress	SECV	Bias
			Calibration	Validation	Calibration	Validation			
OE_1_LEO_1_ (*OEO_1_ adulterated LEO_1_*)	PLSR	Raw	y = 0.9996x + 0.0123	y = 1.0318x − 0.4627	R² = 0.9996	R² = 0.9984	2.14	4.14	1.37
		First derivative.	y = 0.9995x + 0.0139	y = 0.9676x + 0.4399	R² = 0.9995	R² = 0.9950	2.20	4.44	1.50
		Second derivative.	y = 0.9992x + 0.0222	y = 0.9659x + 0.4985	R² = 0.9992	R² = 0.9970	2.40	4.90	1.62
	PCR	Raw	y = 0.9995x + 0.0129	y = 1.0161x − 0.2343	R² = 0.9995	R² = 0.9988	2.13	4.09	0.75
		First derivative.	y = 0.9988x + 0.0337	y = 0.9672x + 0.445	R² = 0.9988	R² = 0.9950	2.19	4.42	1.52
		Second derivative.	y = 0.9995x + 0.0143	y = 0.9761x + 0.1554	R² = 0.9995	R² = 0.9985	0.62	0.72	0.38
OE_2_LEO_2_ (*OEO_2_ adulterated LEO_2_*)	PLSR	Raw	y = 0.9995x + 0.0152	y = 1.0049x + 0.0012	R² = 0.9995	R² = 0.9938	1.58	2.18	1.80
		First derivative.	y = 0.9995x + 0.0149	y = 0.9968x + 0.0991	R² = 0.9995	R² = 0.9977	1.60	2.22	1.17
		Second derivative.	y = 0.9985x + 0.0434	y = 0.9953x + 0.13	R² = 0.9985	R² = 0.9971	1.83	2.92	1.32
	PCR	Raw	y = 0.9993x + 0.0194	y = 1.0049x + 0.0005	R² = 0.9993	R² = 0.9938	1.47	1.92	1.80
		First derivative.	y = 0.9986x + 0.0399	y = 0.9968x + 0.0972	R² = 0.9986	R² = 0.9977	1.54	2.09	1.17
		Second derivative.	y = 0.9983x + 0.0492	y = 0.9953x + 0.1275	R² = 0.9983	R² = 0.9971	1.61	2.26	1.32
OEO_3_LEO_3_ (*OEO_3_ adulterated LEO_3_*)	PLSR	Raw	y = 0.9995x + 0.013	y = 0.9942x + 0.1032	R² = 0.9995	R² = 0.9969	1.92	3.21	0.81
		First derivative.	y = 0.9996x + 0.0123	y = 0.9758x + 0.3435	R² = 0.9996	R² = 0.9964	1.82	2.87	1.13
		Second derivative.	y = 0.9993x + 0.0193	y = 0.9869x + 0.1531	R² = 0.9993	R² = 0.998	1.37	1.70	0.74
	PCR	Raw	y = 0.9979x + 0.0601	y = 0.9942x + 0.1031	R² = 0.9979	R² = 0.9969	1.97	3.40	0.81
		First derivative.	y = 0.998x + 0.0564	y = 0.9757x + 0.344	R² = 0.998	R² = 0.9964	1.54	2.08	1.14
		Second derivative.	y = 0.9979x + 0.0591	y = 0.9669x + 0.4802	R² = 0.9979	R² = 0.9949	1.67	2.43	1.53
BA_1_LEO_1_ (*Benzylalcohol* *adulterated* LEO_1_)	PLSR	Raw	y = 0.9999x + 0.0024	y = 0.9981x + 0.0139	R² = 0.9999	R² = 0.9999	1.24	0.92	0.27
		First derivative.	y = 0.9999x + 0.0035	y = 0.9464x + 0.9899	R² = 0.9999	R² = 0.9985	1.56	2.14	1.90
		Second derivative.	y = 0.9998x + 0.0044	y = 0.9192x + 1.1921	R² = 0.9998	R² = 0.9953	1.84	2.94	3.15
	PCR	Raw	y = 0.9999x + 0.0024	y = 0.9965x + 0.0367	R² = 0.9999	R² = 0.9999	0.43	0.58	0.27
		First derivative.	y = 0.9998x + 0.0043	y = 0.9396x + 1.0674	R² = 0.9998	R² = 0.9981	1.67	2.42	2.17
		Second derivative.	y = 0.9998x + 0.0056	y = 0.9228x + 1.2914	R² = 0.9998	R² = 0.9968	1.89	3.12	2.84
BA_1_LEO_2_ (*Benzylalcohol* *adulterated* LEO)	PLSR	Raw	y = 0.9999x + 0.0028	y = 1.0079x − 0.1288	R² = 0.9999	R² = 0.9998	1.12	1.28	0.33
		First derivative.	y = 0.9999x + 0.0032	y = 0.9646x + 0.6987	R² = 0.9999	R² = 0.9993	1.22	1.44	1.52
		Second derivative.	y = 0.9999x + 0.0036	y = 0.9486x + 0.9728	R² = 0.9999	R² = 0.9986	1.53	2.05	1.80
	PCR	Raw	y = 0.9999x + 0.0028	y = 1.0058x − 0.0992	R² = 0.9999	R² = 0.9998	0.95	1.06	0.31
		First derivative.	y = 0.9999x + 0.0037	y = 0.9578x + 0.806	R² = 0.9999	R² = 0.9990	1.36	1.70	1.47
		Second derivative.	y = 0.9998x + 0.0043	y = 0.9442x + 0.9984	R² = 0.9998	R² = 0.9984	1.60	2.24	2.00
BA_1_LEO_3_ (*Benzylalcohol* *adulterated* LEO_3_)	PLSR	Raw	y = 0.9999x + 0.0030	y = 1.0054x − 0.085	R² = 0.9999	R² = 0.9998	1.13	1.30	0.29
		First derivative.	y = 0.9999x + 0.0028	y = 0.9671x + 0.6537	R² = 0.9999	R² = 0.9994	1.15	1.33	1.12
		Second derivative.	y = 0.9999x + 0.0025	y = 0.9394x + 0.8944	R² = 0.9999	R² = 0.9974	1.53	2.05	2.35
	PCR	Raw	y = 0.9999x + 0.0023	y = 1.0027x − 0.0473	R² = 0.9999	R² = 0.9998	1.01	1.13	0.29
		First derivative.	y = 0.9999x + 0.0031	y = 0.9621x + 0.7607	R² = 0.9999	R² = 0.9992	1.27	1.52	1.29
		Second derivative.	y = 0.9999x + 0.0028	y = 0.9467x + 0.963	R² = 0.9999	R² = 0.9987	1.55	2.11	1.89
IPM_1_LEO_1_ (*isopropylmyristate* *adulterated* LEO_1_)	PLSR	Raw	y = 0.9997x + 0.009	y = 0.9292x + 0.9864	R² = 0.9997	R² = 0.9957	1.98	3.47	2.87
		First derivative.	y = 0.9999x + 0.0034	y = 0.9055x + 1.4095	R² = 0.9999	R² = 0.9923	2.08	3.90	3.78
		Second derivative.	y = 0.9996x + 0.0108	y = 0.9524x + 0.8724	R² = 0.9996	R² = 0.9994	2.00	3.54	2.35
	PCR	Raw	y = 0.9997x + 0.009	y = 0.9307x + 0.9623	R² = 0.9997	R² = 0.9959	1.99	3.49	2.82
		First derivative.	y = 0.9994x + 0.0162	y = 0.9052x + 1.4117	R² = 0.9994	R² = 0.9923	2.11	4.01	3.79
		Second derivative.	y = 0.999x + 0.0281	y = 0.8821x + 1.7718	R² = 0.999	R² = 0.9971	2.33	5.21	4.72
IPM_1_LEO_2_ (*isopropylmyristate* *adulterated* LEO_2_)	PLSR	Raw	y = 0.9997x + 0.0086	y = 0.9328x + 0.9419	R² = 0.9997	R² = 0.9961	1.92	3.21	2.73
		First derivative.	y = 0.9999x + 0.0031	y = 0.9132x + 1.3	R² = 0.9999	R² = 0.9936	2.02	3.62	3.47
		Second derivative.	y = 1x + 0.0008	y = 0.8924x + 1.6224	R² = 1	R² = 0.9994	2.23	4.63	4.31
	PCR	Raw	y = 0.9997x + 0.0086	y = 0.9339x + 0.9249	R² = 0.9997	R² = 0.9962	1.92	3.23	2.70
		First derivative.	y = 0.9995x + 0.0142	y = 0.913x + 1.3013	R² = 0.9995	R² = 0.9935	2.04	3.73	3.48
		Second derivative.	y = 0.9992x + 0.0238	y = 0.8923x + 1.6221	R² = 0.9992	R² = 0.9994	2.27	4.80	4.31
IPM_1_LEO_3_ (*isopropylmyristate* *adulterated* LEO_3_)	PLSR	Raw	y = 0.9995x + 0.0148	y = 0.9171x + 1.1763	R² = 0.9995	R² = 0.9937	2.15	4.20	3.73
		First derivative.	y = 0.9997x + 0.0075	y = 0.8987x + 1.5123	R² = 0.9906	R² = 0.9997	2.15	4.21	4.06
		Second derivative.	y = 0.9998x + 0.0049	y = 0.8795x + 1.8146	R² = 0.9998	R² = 0.9960	2.33	5.15	4.84
	PCR	Raw	y = 0.9995x + 0.0149	y = 0.918x + 1.1602	R² = 0.9938	R² = 0.9995	2.09	3.92	3.34
		First derivative.	y = 0.9992x + 0.0215	y = 0.8984x + 1.5149	R² = 0.9992	R² = 0.9906	2.16	4.28	4.08
		Second derivative.	y = 0.9993x + 0.0194	y = 0.8794x + 1.8155	R² = 0.9993	R² = 0.9959	2.33	5.21	4.85
